# High prevalence of Cfr-producing *Staphylococcus* species in retail meat in Guangzhou, China

**DOI:** 10.1186/1471-2180-14-151

**Published:** 2014-06-09

**Authors:** Zhen-Ling Zeng, Hong-Kun Wei, Jing Wang, Da-Chuan Lin, Xiao-Qin Liu, Jian-Hua Liu

**Affiliations:** 1 College of Veterinary Medicine, National Reference Laboratory of Veterinary Drug Residues (SCAU), South China Agricultural University, Guangzhou, China; 2 Department of Applied Biology and Chemical Technology, The Hong Kong Polytechnic University, Hong Kong, China

**Keywords:** Plasmids, Linezolid, *Staphylococcus spp*, Food safety, Resistance epidemiology

## Abstract

**Background:**

The emergence and wide distribution of the transferable gene for linezolid resistance, *cfr*, in staphylococci of human and animal origins is of great concern as it poses a serious threat to the public health. In the present study, we investigated the emergence and presence of the multiresistance gene, *cfr*, in retail meat sourced from supermarkets and free markets of Guangzhou, China.

**Results:**

A total of 118 pork and chicken samples, collected from Guangzhou markets, were screened by PCR for *cfr*. Twenty-two *Staphylococcus* isolates obtained from 12 pork and 10 chicken samples harbored *cfr*. The 22 *cfr*-positive staphylococci isolates, including *Staphylococcus equorum* (n = 8), *Staphylococcus simulans* (n = 7), *Staphylococcus cohnii* (n = 4)*,* and *Staphylococcus sciuri* (n = 3), exhibited 17 major *SmaI* pulsed-field gel electrophoresis (PFGE) patterns. In 14 isolates, *cfr* was located on the plasmids. Sequence analysis revealed that the genetic structures (including Δ*tnpA* of Tn*558*, IS*21-558*, Δ*tnpB*, and *tnpC* of Tn*558*, *orf138*, *fexA*) of *cfr* in plasmid pHNTLD18 of a *S. sciuri* strain and in the plasmid pHNLKJC2 (including *rep*, Δ*pre/mob, cfr*, *pre/mob* and partial *ermC*) of a *S. equorum* strain were identical or similar to the corresponding regions of some plasmids in staphylococcal species of animal and human origins.

**Conclusions:**

To the best of our knowledge, this is the first study to report the presence of the multiresistance gene, *cfr,* in animal meat. A high occurrence of *cfr* was observed in the tested retail meat samples. Thus, it is important to monitor the presence of *cfr* in animal foods in China.

## Background

Linezolid is considered to as the last treatment option for infections caused by methicillin-resistant *Staphylococcus aureus* (MRSA), vancomycin-resistant *Enterococci* and penicillin-resistant *Streptococcus*[1]. Mutations in the drug target site (23S rRNA or ribosomal proteins L3 and L4) are the most common mechanisms of linezolid resistance. Due to the low frequency of target mutation, the frequency of linezolid resistance is also relatively low [2]. However, emergence of the transferable linezolid resistance gene, *cfr,* in clinical isolates poses a challenge in linezolid treatment. *cfr* gene encodes an RNA methyltransferase, which modifies the adenine residue at position 2503 of the 23S rRNA gene and thereby confers resistance to phenicols, lincosamides, oxazolidinones, pleuromutilins, and streptogramin A antibiotics (the PhLOPS_A_ phenotype) as well as decreases susceptibility to the 16-membered macrolides spiramycin and josamysin [3-5].

Since its first detection from a bovine *Staphylococcus sciuri* isolate in 1997 [6], *cfr* has been globally transmitted among different bacteria, such as *Staphylococcus* spp., *Enterococcus* spp., *Macrococcus* spp., *Jeotgalicoccus* spp., *Streptococcus suis*, *Escherichia coli*, *Bacillus* spp., *Proteus vulgaris*[7,8]. This gene is widely distributed in the isolates of both human and animal origin, especially in China [8]. A recent study has described this gene in farm environments [9]. However, there has been no study on the distribution of *cfr* in retail meat. In the present study, we investigated the presence and the genetic background of this multiresistance gene in retail meat samples sourced from supermarkets and free markets of Guangzhou, China.

## Results

### Identification of *cfr*-positive *Staphylococcus* isolates

Of the 118 retail meat samples tested, a total of 22 *cfr*-positive *Staphylococcus* isolates were detected in 12 pork samples and 10 chicken samples. The 22 *cfr*-positive staphylococcal isolates included *Staphylococcus equorum* (n = 8), *Staphylococcus simulans* (n = 7), *Staphylococcus cohnii* (n = 4), and *Staphylococcus sciuri* (n = 3). In addition, one *cfr*-positive *Macrococcus caseolyticus* isolate was obtained from a chicken sample. In total, 15.8% and 26.2% pork and chicken samples carried *cfr*-positive isolates, respectively.

### Clonal analysis of *cfr*-positive staphylococci and location of *cfr*

Pulsed-field gel electrophoresis (PFGE) of 22 *cfr*-positive staphylococci revealed 17 major *SmaI* PFGE patterns (Table 1). Eight *S. equorum* isolates showed five different PFGE patterns, with two chicken strains from the same market presenting indistinguishable patterns. Six distinct PFGE patterns were identified for the seven *S. simulans* isolates, with only two pork isolates from different markets presenting similar PFGE patterns. For the four *S. cohnii* isolates, three PFGE patterns were identified, with two pork isolates from the same market presenting identical patterns. Each of the three *S. sciuri* isolates exhibited distinct PFGE patterns. In summary, most of the *cfr*-positive staphylococcal isolates were genetically distinct, but a clonal transfer of *cfr*-positive staphylococcal isolates had occurred either in the same or among different markets.

**Table 1:** **Characteristics of *cfr*-carrying isolates and transformants**

**Isolate**	**Staphylococcal species**	**Origin**	**Market**	**PFGE type**^**a**^	**Location of *cfr***^**b**^	**MIC values of antimicrobial agents (mg/L)**^**c**^						**Other resistance patterns**^**d**^
						**CHL**	**FFC**	**CLR**	**TIA**	**VAL**	**LZD**	
TDP5	*S. cohnii*	Pork	1	C	P	16	>64	>64	128	64	2	OXA, CIP, GEN, ERY, TET
TDPJC2	*S. cohnii*	Chicken	1	P	ND	32	32	>64	64	0.5	2	OXA, CIP, ERY
TYT5	*S. cohnii*	Pork	3	F	P	32	32	64	128	64	2	TET
TYT7	*S. cohnii*	Pork	3	F	P	16	>64	>64	64	16	2	OXA, CIP, GEN, ERY
TDP9	*S. equorum*	Pork	1	D	P	32	>64	>64	>128	>64	8	OXA, GEN, ERY, TET
TDPJC9	*S. equorum*	Chicken	1	J	P	16	64	>64	128	2	4	OXA, GEN, ERY, TET
TLD18	*S. equorum*	Pork	2	L1	P	16	>64	>64	>128	64	8	OXA, GEN, ERY, TET
TLDJC5	*S. equorum*	Chicken	2	L2	P	64	32	>64	>128	16	4	OXA, CIP, GEN, ERY, RIF, TET
TLDJC9	*S. equorum*	Chicken	2	N	P	32	64	>64	>128	2	4	OXA, CIP, GEN, ERY, RIF, TET
TLH5	*S. equorum*	Pork	4	L3	ND	16	>64	>64	128	8	4	OXA, CIP, GEN, ERY, TET
TYTJC3	*S. equorum*	Chicken	3	I	ND	16	32	>64	>128	4	4	ERY, TET
TYTJC8	*S. equorum*	Chicken	3	I	ND	16	32	64	64	16	2	OXA, CIP, GEN, ERY
TDPJC13	*S. sciuri*	Chicken	1	E	P	64	64	>64	>128	32	4	OXA, CIP, GEN, TET
TDPJC5	*S. sciuri*	Chicken	1	R	ND	32	>64	>64	>128	>64	16	OXA, GEN, ERY, TET
TLKJC2	*S. sciuri*	Chicken	6	Q	P	16	>64	>64	>128	16	8	OXA, CIP, GEN, ERY, TET
TDP12	*S. simulans*	Pork	1	A	ND	>64	64	64	64	16	4	OXA, CIP, GEN, ERY, RIF
TDP24	*S. simulans*	Pork	1	B	ND	32	>64	>64	64	2	4	TET
THTJC2	*S. simulans*	Chicken	5	O	P	64	32	>64	>128	4	4	OXA, CIP, GEN, ERY, RIF
TLD12	*S. simulans*	Pork	2	K	P	>64	>64	>64	>128	64	8	OXA, CIP, GEN, ERY, RIF, TET
TLD20	*S. simulans*	Pork	2	M	P	>64	>64	>64	128	32	4	OXA, CIP, GEN, ERY, RIF, TET
TLD22	*S. simulans*	Pork	2	G2	P	16	>64	>64	128	8	8	CIP, GEN, ERY, TET
TYT6	*S. simulans*	Pork	3	G1	ND	16	>64	>64	>128	64	4	OXA, ERY, TET
Recipient RN4220	*S. aureus*					4	4	0.25	0.5	0.25	1	ND
RN4220-pHNLKJC2	*S. aureus*					32	64	16	16	8	4	ND
DH5α	*E. coli*					4	4	-	-	-	-	ND
DH5*α*-pUC18-cfr	*E. coli*					8	8	-	-	-	-	ND
ATCC 29213	*S. aureus*					2	2	0.12	0.5	0.06	1	

^a^Patterns that differed from pattern A by six or more bands were considered to represent different strains. Patterns that differed by fewer than six bands were considered to represent subtypes within the main group (e.g.,L1, L2).

^b^P, plasmid; ND, not determined.

^c^CHL, chloramphenicol; FFC, florfenicol; CLR, clindamycin; TIA, tiamulin; VAL, valnemulin; LZD, linezolid. MIC was not measured because of known intrinsic resistance or naturally high MICs.

^d^The results were interpreted according to Eucast breakpoints ( http://www.eucast.org/clinical_breakpoints/). OXA, oxacillin; CIP, ciprofloxacin; GEN, gentamycin; ERY, erythromycin; RIF, rifamycin; TET, tetracycline. All isolates were susceptible to vancomycin. ND, not determined.

Results of Southern blotting indicated that 14 isolates harbored *cfr* in their plasmid DNA (Table 1). The remaining eight isolates appeared to carry *cfr* in their genomic DNA; however, this assumption needs to be further confirmed by S1-PFGE. Only one *cfr*-carrying plasmid (designated as pHNLKJC2) that originated from a chicken isolate, TLKJC2, was transformed into *Staphylococcus aureus* RN4220. The transformant was confirmed by polymerase chain reaction (PCR) for *cfr*; it showed the same PFGE pattern as that of *Staphylococcus aureus* RN4220.

### Antimicrobial susceptibility of *cfr*-positive *Staphylococcus* isolates and the transformants

All of the 22 *cfr*-positive staphylococcal isolates had elevated minimum inhibitory concentrations (MICs) against chloramphenicol (16 to > 64 mg/L), florfenicol (32 to >64 mg/L), clindamycin (≥64 mg/L), tiamulin (64 to > 128 mg/L), valnemulin (0.5 to >64 mg/L), and linezolid (2 to 16 mg/L) (Table 1). In addition, 18, 14, 13, 17, 6, and 17 isolates exhibited resistance to oxacillin, ciprofloxacin, gentamicin, erythromycin, rifampicin, and tetracycline, respectively. All isolates were found to be susceptible to vancomycin.

Compared with *S. aureus* RN4220, the transformant carrying pHNLKJC2 had elevated MICs against chloramphenicol (8-fold), florfenicol (16-fold), clindamycin (64-fold), tiamulin (32-fold), valnemulin (32-fold), and linezolid (4-fold) (Table 1), supporting the presence and the functional activity of *cfr*. In addition, the transformant carrying pUC18-cfr exhibited 2-fold-elevated MICs for chloramphenicol and florfenicol as compared to *E. coli* DH5α.

### Analysis of the genetic environment of *cfr* in the plasmid pHNTLD18 and pHNLKJC2

**Figure 1:**
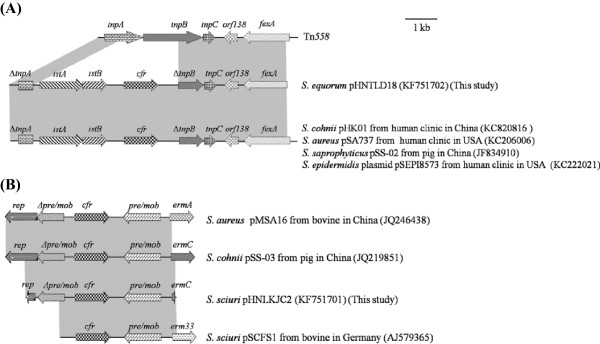
**Genetic environment of *cfr *in plasmids pHNTLD18 and pHNLKJC2 and comparison with other similar plasmids.** The arrows indicate the positions and directions of the transcription of the genes. Regions of >98% homology are shaded in grey. Δ indicates a truncated gene. **A**. genetic environment of *cfr* in pHNTLD18; **B**. genetic environment of *cfr* in pHNLKJC2.

Southern blotting confirmed that, in *Staphylococcus equorum* TLD18, *cfr* was located on a plasmid designed as pHNTLD18. An approximately 5.7-kb *EcoRI* fragment containing *cfr* was cloned and sequenced. A Tn*558* variant was identified on the plasmid pHNTLD18, in which parts of the Tn*558*-associated transposase genes *tnp*A and *tnp*B were replaced by a *cfr-*carrying segment and the insertion sequence IS*21-558* (Figure 1A). Another resistance gene, *fexA*, encoding an exporter that mediates the active efflux of phenicols, was found to be located downstream of Tn*558*.

The sequences 1,926-bp upstream and 2,659-bp downstream of *cfr* on the plasmid pHNLKJC2 were obtained by primer walking. Basic local alignment search tool (BLAST) analysis of these sequences revealed a 3′-truncated segment of the gene *pre/mob* upstream of *cfr*. Further upstream, an incomplete *rep* gene was detected. Analysis of the region downstream of *cfr* revealed the presence of a complete *pre/mob* gene. Immediately downstream of the *pre/mob* gene, an incomplete macrolide-lincosamide-streptogramin B (MLS_B_) resistance gene *ermC* was detected (Figure 1B).

## Discussion

Lack of previous studies on the distribution of the multiresistance gene *cfr* among staphylococci in retail meat led us to screen 118 meat samples for the same. In our analysis, *cfr* was detected in 22 samples. The detection rate was 18.6%, which is higher than the detection rates of food animal samples in China [10,11]. The low fitness cost of *cfr* acquisition observed in staphylococcal isolates may account for the persistence of this multiresistance gene in retail meat even in the absence of an antimicrobial selection pressure [12]. The high detection rate found in this study suggested that *cfr* may be widely disseminated among staphylococci in the meats sold in China, increasing the possibility of this gene entering the food chain.

In this study, *S. equorum* (n = 8) was the predominant species among the 22 *cfr*-carrying isolates obtained from animal food sources. To the best of our knowledge, this is the first report of *cfr* in *S. equorum. S. equorum* was originally isolated from the skin of horses and was later found to be the predominant species of staphylococci in sausages and cheese samples [13-17]. *S. equorum* is used as one of the starter cultures in the preparation of smear-ripened cheese and cured meats such as sausages [15,16]. Since *S. equorum* present in retail meats has rare chances of coming in contact with antimicrobial agents, the origin and high prevalence of *cfr* in *Staphylococcus equorum* is intriguing.

The *cfr*-carrying segment (including *rep*, *Δpre/mob, cfr*, *pre/mob* and partial *ermC*) on the plasmid pHNLKJC2 from the chicken meat strain *S. sciuri* TLKJC2, was found to be similar to the corresponding plasmid regions from different staphylococcal species such as the plasmid pSS-03 (accession number JQ219851) from a bovine *S. cohnii* strain and the plasmid pMSA16 (accession number JQ246438) from a bovine MRSA ST9 strain in China (Figure 1B) [10,18]. In addition, this *cfr*-carrying segment also showed high nucleotide sequence identity (98%) to the corresponding region of plasmid pSCFS1 (accession number AJ579365) from a bovine *S. sciuri* in Germany [19]. The *cfr*-carrying segment (including *ΔtnpA* of Tn*558*, IS*21-558*; *ΔtnpB*; and *tnpC* of Tn*558*, *orf138*, *fexA*) on the plasmid pHNTLD18 from the pork strain *S. equorum* TLD18 was identical to the corresponding segment of the plasmid pHK01 (accession number KC820816) found in *S. cohnii* from human in China [20], the plasmid pSA737 (accession number KC206006) extracted from a human clinical MRSA strain and the plasmid pSEPI8573 (accession number KC222021) from a human clinical *S. epidermidis* strain in the United States [21], and the plasmid pSS-02 (accession number JF834910) obtained from a porcine *S. saprophyticus* strain in China(Figure 1A) [10]. These results indicated that the horizontal transfer mediated by mobile genetic elements such as plasmids and insertion sequences may contribute to the spread of *cfr* and suggested that it is possible to transfer *cfr* via mobile genetic elements from staphylococcal isolates of animal origin to the bacterial strains in the human body through meat consumption, posing a serious threat to the public health.

The MICs of the *cfr*-positive staphylococci indicated multiresistance phenotype in these strains other than the PhLOPS_A_ phenotype, suggesting limited therapeutic options to control these *cfr*-carrying staphylococci. Most of the *cfr*-positive staphylococcal isolates showed low-level linezolid resistance with MIC values ranging from 4 to 16 mg/L; this result is in agreement with previously reported linezolid MICs among *cfr*-carrying staphylococci from farm animals and humans [10,11,22]. In addition, five of the *cfr*-positive isolates had linezolid MIC values of 2 mg/L, which is the same as the typical linezolid MIC_90_ value and not consistent with MIC value shifts observed for isogenic *cfr*-negative/positive staphylococcal strain pairs [23]. This finding indicated that *cfr*, although present, is not functional or is partially silenced in some way [24]. Although *Staphylococcus* strains isolated from meat samples showed low-level of linezolid resistance in the present study, emergence of the multiresistance gene *cfr* in meat poses a potentially significant threat to the public health, considering that the *cfr*-mediated linezolid resistance can rapidly and widely spread among different bacterial species.

## Conclusions

To the best of our knowledge, this is the first study to report a surprisingly high occurrence of *cfr* in retail meat samples in Chinese markets. Animal meat harboring bacteria containing the transmissible *cfr* would be a serious threat to the public health as these bacteria may act as reservoirs for spreading *cfr* to bacteria that infect humans, particularly in environments with a large microbial community. Recently, *cfr* was detected in human isolates in China [20,25]. Thus, more attention needs to be paid to the possibility that *cfr* can find its way through the food chain to commensal or pathogenic bacteria of humans. Considering that a limited number of meat samples were used and that to from only one city in China, the results of the present study regarding dissemination of *cfr* among staphylococcal species from animal food sources in China is not conclusive. Thus, continuing the surveillance of *cfr* gene in meat distributed in China is critical to limit its dissemination, which could potentially threaten the human health.

## Methods

### Sample collection, identification of species, and *cfr* detection

In February 2012, 72 pork samples and 46 chicken samples were collected from five free markets and one supermarket in Guangzhou. The meat samples were incubated in Luria–Bertani (LB) broth for enrichment. Then, the cultured broth was streaked onto selective media plates of Baird–Parker agar supplemented with 10 mg/L florfenicol. One isolate per sample was selected for further analysis. Whole-cell DNA was prepared according to a previously described protocol [26]. The presence of *cfr* was screened by PCR with previously described primers [5]. Species identification of the *cfr*-carrying strains was performed by the API-Staph System (bioMérieux, France) and further confirmed by 16S rRNA sequencing [27].

### Molecular typing and transformation

PFGE of all *cfr*-positive *Staphylococcus* isolates was performed by using the CHEF Mapper System (Bio-Rad Laboratories, Hercules, CA), according to the previously described protocol [10]. All the plugs of genomic DNA were digested with *SmaI* (TaKaRa Biotechnology, Dalian, China). The PFGE patterns were interpreted according to the criteria described by Tenover et al. [28]. The location of *cfr* was determined by Southern blotting.

*Cfr-*carrying plasmids of the isolates were extracted by using the QIAGEN Plasmid DNA Midi Kit (Qiagen, Hilden, Germany) and then transferred into *S. aureus* RN4220 by electrotransformation, as described previously [29]. The transformants were selected on media supplemented with 10 mg/L florfenicol, screened for *cfr* by PCR amplification, and confirmed by PFGE.

### Antimicrobial susceptibility testing

The MIC values of all *cfr*-positive original *Staphylococcus* isolates and transformants were determined by the broth microdilution method, according to the recommendations specified in CLSI documents M100-S22 [30]. The results were interpreted according to Eucast breakpoints ( http://www.eucast.org/clinical_breakpoints/). Isolates with an MIC of ≥16 mg/L were tentatively considered to be florfenicol-resistant [26]. The reference strain *S. aureus* ATCC 29213 was used for quality control.

### Cloning and sequencing of the regions flanking *cfr*

The regions flanking *cfr* in the transformant obtained from the isolate TLKJC2 were determined by PCR mapping. The plasmid DNA of the isolate TLD18 was extracted and digested with *EcoRI*. The digested fragments were cloned into the pUC18 vector, and the recombinant plasmid (designated as pUC18-cfr) was introduced into *Escherichia coli* DH5α with subsequent selection for the transformant (designated as *E. coli* DH5α- pUC18-cfr) on media supplemented with 10 mg/L florfenicol. The approximately 5.7-kb segment in pUC18-cfr, including *cfr* and its flanking regions, was sequenced by primer walking. The DNA sequences were compared to those deposited in GenBank using the BLAST program ( http://www.ncbi.nlm.nih.gov/BLAST).

### Nucleotide sequence accession number

The nucleotide sequences of *cfr*-containing fragments of plasmids pHNLKJC2 and pHNTLD18 have been deposited in the GenBank under the accession numbers KF751701 and KF751702, respectively.

## Competing interests

The authors declare that they have no competing interests.

## Authors’ contributions

JHL, ZLZ, and DCL conceived the study. HKW, JW, ZLZ, and XQL carried out the experiments, ZLZ, HKW, and WJ wrote the manuscript. JHL revised the manuscript. All authors have read and approved the final manuscript.

## References

[B1] Bozdogan B Appelbaum PC Oxazolidinones: activity, mode of action, and mechanism of resistance Int J Antimicrob Agents 2004 23 113 119 15013035 10.1016/j.ijantimicag.2003.11.003

[B2] Shaw KJ Barbachyn MR The oxazolidinones: past, present, and future Ann NY Acad Sci 2011 1241 48 70 22191526 10.1111/j.1749-6632.2011.06330.x

[B3] Kehrenberg C Schwarz S Jacobsen L Hansen LH Vester B A new mechanism for chloramphenicol, florfenicol and clindamycin resistance: methylation of 23S ribosomal RNA at A2503 Mol Microbiol 2005 57 1064 1073 16091044 10.1111/j.1365-2958.2005.04754.x

[B4] Long KS Poehlsgaard J Kehrenberg C Schwarz S Vester B The Cfr rRNA methyltransferase confers resistance to phenicols, lincosamides, oxazolidinones, pleuromutilins, and streptogramin A antibiotics Antimicrob Agents Chemother 2006 50 2500 2505 16801432 10.1128/AAC.00131-06 PMC1489768

[B5] Smith LK Mankin AS Transcriptional and translational control of the mlr operon, which confers resistance to seven classes of protein synthesis inhibitors Antimicrob Agents Chemother 2008 52 1703 1712 18299405 10.1128/AAC.01583-07 PMC2346656

[B6] Schwarz S Werckenthin C Kehrenberg C Identification of a plasmid-borne chloramphenicol-florfenicol resistance gene in *Staphylococcus sciuri* Antimicrob Agents Chemother 2000 44 2530 2533 10952608 10.1128/aac.44.9.2530-2533.2000 PMC90098

[B7] Wang Y Li D Song L Liu Y He T Liu H Wu C Schwarz S Shen J First report of the multiresistance gene *cfr* in *streptococcus suis* Antimicrob Agents Chemother 2013 57 4061 4063 23733472 10.1128/AAC.00713-13 PMC3719703

[B8] Shen J Wang Y Schwarz S Presence and dissemination of the multiresistance gene *cfr* in Gram-positive and Gram-negative bacteria J Antimicrob Chemother 2013 68 1697 1706 23543608 10.1093/jac/dkt092

[B9] Liu Y Wang Y Schwarz S Li Y Shen Z Zhang Q Wu C Shen J Transferable multiresistance plasmids carrying *cfr* in *Enterococcus spp.* from swine and farm environment Antimicrob Agents Chemother 2013 57 42 48 23070165 10.1128/AAC.01605-12 PMC3535926

[B10] Wang Y Zhang W Wang J Wu C Shen Z Fu X Yan Y Zhang Q Schwarz S Shen J Distribution of the multidrug resistance gene *cfr* in *Staphylococcus* species isolates from swine farms in China Antimicrob Agents Chemother 2012 56 1485 1490 22183168 10.1128/AAC.05827-11 PMC3294914

[B11] Wang Y He T Schwarz S Zhao Q Shen Z Wu C Shen J Multidrug resistance gene *cfr* in methicillin-resistant coagulase-negative staphylococci from chickens, ducks, and pigs in China Int J Med Microbiol 2013 303 84 87 23337100 10.1016/j.ijmm.2012.12.004

[B12] LaMarre JM Locke JB Shaw KJ Mankin AS Low fitness cost of the multidrug resistance gene *cfr* Antimicrob Agents Chemother 2011 55 3714 3719 21646483 10.1128/AAC.00153-11 PMC3147626

[B13] Schleifer KH Kilpper Baltz R Devriese LA *Staphylococcus arletae* sp.nov., S. *equorum* sp. nov. and *S. kloosii* sp. nov.: three new coagulase-negative, novobiocin-resistant species from animals Syst Appl Microbiol 1984 5 501 509

[B14] Corbière Morot-Bizot S Leroy S Talon R Staphylococcal community of a small unit manufacturing traditional dry fermented sausages Int J Food Microbiol 2006 108 210 217 16488037 10.1016/j.ijfoodmicro.2005.12.006

[B15] Mauriello G Casaburi A Blaiotta G Villani F Isolation and technological properties of coagulase negative staphylococci from fermented sausages of Southern Italy Meat Sci 2004 67 149 158 22061128 10.1016/j.meatsci.2003.10.003

[B16] Bockelmann W Development of defined surface starter cultures for the ripening of smear cheeses Int Dairy J 2002 12 123 131

[B17] Irlinger F Morvan A El Solh N Bergere JL Taxonomic characterization of coagulase-negative staphylococci in ripening flora from traditional French cheeses Syst Appl Microbiol 1997 20 319 328

[B18] Wang X Zhang W Schwarz S Yu S Liu H Si W Zhang R Liu S Methicillin-resistant *Staphylococcus aureus* ST9 from a case of bovine mastitis carries the genes *cfr* and *erm* (A) on a small plasmid J Antimicrob Chemother 2012 67 1287 1289 22334606 10.1093/jac/dks028

[B19] Kehrenberg C Ojo KK Schwarz S Nucleotide sequence and organization of the multiresistance plasmid pSCFS1 from *Staphylococcus sciuri* J Antimicrob Chemother 2004 54 936 939 15471995 10.1093/jac/dkh457

[B20] Chen H Wu W Ni M Liu Y Zhang J Xia F He W Wang Q Wang Z Cao B Linezolid-resistant clinical isolates of *enterococci* and *Staphylococcus cohnii* from a multicentre study in China: molecular epidemiology and resistance mechanisms Int J Antimicrob Agents 2013 42 317 321 23880167 10.1016/j.ijantimicag.2013.06.008

[B21] Mendes RE Deshpande LM Bonilla HF Schwarz S Huband MD Jones RN Quinn JP Dissemination of a pSCFS3-like *cfr*-carrying plasmid in *Staphylococcus aureus* and *Staphylococcus epidermidis* Clinical Isolates Recovered from Hospitals in Ohio Antimicrob Agents Chemother 2013 57 2923 2928 23571552 10.1128/AAC.00071-13 PMC3697371

[B22] Mendes RE Hogan PA Streit JM Jones RN Flamm RK Zyvox(R) Annual appraisal of potency and spectrum (ZAAPS) program: report of linezolid activity over 9 years (2004–12) J Antimicrob Chemother 2014 69 1582 1588 24468866 10.1093/jac/dkt541

[B23] Locke JB1 Morales G Hilgers M GC K Rahawi S Jose Picazo J Shaw KJ Stein JL Elevated linezolid resistance in clinical *cfr*-positive *Staphylococcus aureus* isolates is associated with co-occurring mutations in ribosomal protein L3 Antimicrob Agents Chemother 2010 54 5352 5355 20837755 10.1128/AAC.00714-10 PMC2981277

[B24] Liu Y Wang Y Schwarz S Wang S Chen L Wu C Shen J Investigation of a multiresistance gene *cfr* that fails to mediate resistance to phenicols and oxazolidinones in *Enterococcus faecalis* J Antimicrob Chemother 2014 69 892 898 24272266 10.1093/jac/dkt459

[B25] Cui L Wang Y Li Y He T Schwarz S Ding Y Shen J Lv Y Cfr-mediated linezolid-resistance among methicillin-resistant coagulase-negative staphylococci from infections of humans PLoS One 2013 8 e57096 23437319 10.1371/journal.pone.0057096 PMC3577776

[B26] Kehrenberg C Schwarz S Distribution of florfenicol resistance genes *fexA* and *cfr* among chloramphenicol-resistant *Staphylococcus* isolates Antimicrob Agents Chemother 2006 50 1156 1163 16569824 10.1128/AAC.50.4.1156-1163.2006 PMC1426988

[B27] Kim TW Kim SE Park CS Identification and distribution of *Bacillus* species in doenjang by whole-cell protein patterns and 16S rRNA gene sequence analysis J Microbiol Biotechnol 2010 20 1210 1214 20798584 10.4014/jmb.1002.02008

[B28] Tenover FC Arbeit RD Goering RV Mickelsen PA Murray BE Persing DH Swaminathan B Interpreting chromosomal DNA restriction patterns produced by pulsed-field gel electrophoresis: criteria for bacterial strain typing J Clin Microbiol 1995 33 2233 2239 7494007 10.1128/jcm.33.9.2233-2239.1995 PMC228385

[B29] Schenk S Laddaga RA Improved method for electroporation of *Staphylococcus aureus* FEMS Microbiol Lett 1992 94 133 138 1521761 10.1016/0378-1097(92)90596-g

[B30] CLSI Performance Standards for Antimicrobial Susceptibility Testing; Twenty-Second Informational Supplement CLSI document M100-S22 2012 Wayne, PA: Clinical and Laboratory Standards Institute

